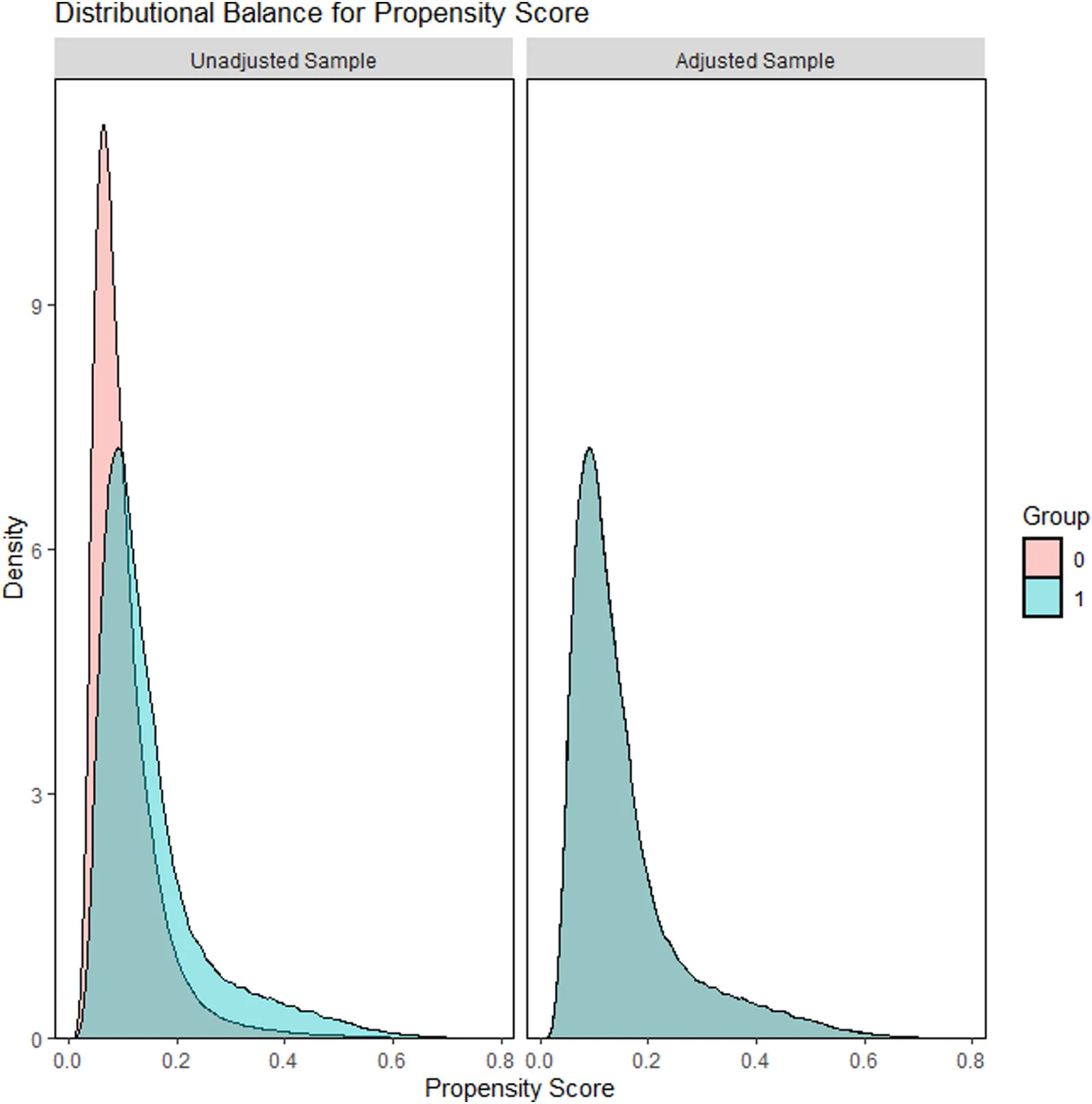# Pre-admission prescription of selective serotonin reuptake inhibitors and mortality in critical illness: a national cohort study from 2020 to 2023

**DOI:** 10.1016/j.aicoj.2026.100161

**Published:** 2026-09-17

**Authors:** Tak Kyu Oh, In-Ae Song

**Affiliations:** a Department of Anesthesiology and Pain Medicine, Seoul National University Bundang Hospital, Bundang‑gu, Seongnam 13620, Republic of Korea; b Department of Anesthesiology and Pain Medicine, College of Medicine, Seoul National University, Jongno‑gu, Seoul 03080, Republic of Korea

**Keywords:** Selective serotonin reuptake inhibitors, Critical illness, Intensive care unit, Mortality, Psychiatric morbidity

## Abstract

**Background:**

We aimed to determine whether selective serotonin reuptake inhibitor (SSRI) prescription within 30 days before intensive care unit (ICU) admission was associated with 30-day and 90-day mortality in critically ill adults, and to evaluate effect modification by underlying psychiatric disorders.

**Methods:**

We conducted a nationwide retrospective cohort study of adults with a first ICU admission in South Korea from 2020 through 2023. Pre-admission SSRI prescription was defined using prescription supply records during the 30 days before ICU admission. Propensity score matching and Cox proportional hazards models were used to evaluate 30-day and 90-day mortality.

**Results:**

Pre-admission SSRI prescription was associated with lower 30-day and 90-day mortality after propensity score matching. Similar associations were observed in the full cohort and when prescription coverage included the day immediately before ICU admission. The associations appeared stronger among patients with documented psychiatric disorders.

**Conclusions:**

Pre-admission SSRI prescription was associated with lower short- and intermediate-term mortality in critically ill adults. These observational findings do not establish a causal protective effect and should be interpreted in light of potential residual confounding.

**Trial registration:**

None.

## Highlights

• Pre-admission SSRI prescription was associated with lower 30-day and 90-day mortality.
• Similar associations were observed when prescription coverage included the day before ICU admission.
• Associations appeared stronger among patients with documented psychiatric disorders.
• These observational findings do not establish a causal or therapeutic effect of SSRIs.

## Background

Critical illness remains a leading cause of mortality worldwide despite advances in supportive care, and the search for adjunctive therapies to improve outcomes in the intensive care unit (ICU) is ongoing [1]. Selective serotonin reuptake inhibitors (SSRIs) are widely prescribed for depression and anxiety disorders, but emerging evidence suggests that they may exert immunomodulatory and metabolic effects that could influence survival in severe infections and critical illness [2]. In preclinical models, SSRIs such as fluoxetine have been shown to upregulate anti‐inflammatory cytokines, preserve organ function, and reduce mortality in experimental sepsis [3].

Human evidence regarding the association between SSRI use and outcomes in critical illness remains limited and inconsistent. An earlier retrospective ICU study reported increased mortality among patients receiving SSRIs before ICU admission [4]. In contrast, several studies conducted during the COVID-19 pandemic, including randomized and observational investigations of fluvoxamine, fluoxetine, and other antidepressants, suggested potential clinical benefits in selected populations [[5], [6], [7], [8], [9]]. However, these findings arose largely from disease-specific populations and may not be generalizable to the broader, heterogeneous ICU population. Whether pre-admission SSRI prescription is associated with mortality among critically ill adults across a wide range of underlying conditions therefore remains uncertain.

In this nationwide retrospective cohort study using South Korea’s National Health Insurance Service (NHIS) database from 2020 to 2023, we therefore sought to determine whether SSRI prescription within 30 days before ICU admission was associated with 30-day and 90-day mortality among critically ill adults. By applying propensity score (PS) matching, Cox proportional hazards models, and additional sensitivity analyses, we aimed to evaluate the association between pre-admission SSRI prescription and short- and intermediate-term mortality in real-world critical care practice.

## Methods

### Study design and ethical approval

This study was a retrospective cohort analysis based on nationwide administrative healthcare data and was prepared in accordance with the Strengthening the Reporting of Observational Studies in Epidemiology (STROBE) guidelines [10]. The study protocol received ethical clearance from the Institutional Review Board of Seoul National University Bundang Hospital (X-2410-930-905) and the NHIS (NHIS-2025-04-1-108), which also authorized data access. As all personal identifiers were removed and the dataset was originally compiled for administrative and reimbursement purposes, the requirement for informed consent was waived in line with Korean law and the ethical standards outlined in the Declaration of Helsinki (1975, revised 2008).

### Data source

We obtained data from the NHIS, which operates South Korea’s compulsory, single-payer national health insurance system. This comprehensive database includes longitudinal patient-level information on diagnoses, procedures, prescriptions, mortality, and demographic characteristics. Diagnoses are recorded using codes from the International Classification of Diseases, 10th Revision (ICD-10). Although the majority of physicians practice in private institutions, standardized reporting protocols and government oversight ensure high-quality data across healthcare providers in South Korea [11]. Key demographic, insurance, prescription, diagnostic, procedural, disability registration, and mortality variables are systematically collected by the NHIS for reimbursement, eligibility assessment, and national health administration. In South Korea, individuals with disabilities are registered in the Korean National Disability Registration System after formal assessment by qualified specialists in the relevant clinical field, generally after a sufficient duration of impairment, and disability severity is officially graded within this national registration framework [12].

### Study population

We identified all adult patients aged ≥18 years who were admitted to an ICU in South Korea between January 1, 2020, and December 31, 2023. The date of ICU admission was defined as the index date. Patients with missing information on age or sex were excluded, as were pediatric patients. For patients with more than one ICU admission during the study period, only the first ICU admission was retained in the primary analysis to avoid within-person correlation and to ensure a uniform assessment of pre-ICU SSRI prescription before the index admission.

### SSRI prescription

Prescription records for selective serotonin reuptake inhibitors (SSRIs) were extracted from the NHIS database, where all reimbursed prescriptions must be registered. The SSRIs extracted for this study were fluoxetine, paroxetine, sertraline, citalopram, escitalopram, and fluvoxamine, based on NHIS prescription drug codes. We defined SSRI prescription as having at least one SSRI prescription with a supply period overlapping any day within the 30-day period before the index date, defined as the date of ICU admission. Patients meeting this criterion comprised the SSRI prescription group, whereas those without prescription supply overlapping this 30-day pre-ICU window comprised the no SSRI prescription group. Thus, patients whose previous SSRI prescription supply ended more than 30 days before ICU admission were classified in the no SSRI prescription group.

### Study endpoint

We defined 30-day mortality and 90-day mortality as death occurring within 30 days and within 90 days, respectively, from the date of ICU admission. These two measures served as our primary endpoints. Exact dates of death—including those occurring after hospital discharge—were obtained from the NHIS database, which maintains comprehensive records for all enrollees. Mortality information was available through December 31, 2024, providing at least 365 days of mortality ascertainment for all patients included through December 31, 2023 and ensuring complete ascertainment of the prespecified 30-day and 90-day mortality outcomes. For time-to-event analyses, follow-up time was calculated from the index date to the date of death or censoring at 30 days and 90 days, respectively.

### Covariates

We adjusted for demographic, socioeconomic, comorbidity, acute clinical, and institutional variables. First, demographic factors included patient age as a continuous variable and sex at the time of ICU admission. Socioeconomic status was assessed using area of residence (urban—defined as the capital and other major cities—versus rural) and household income, categorized into four quartiles plus a Medical Aid group for individuals exempt from NHIS premiums. To capture underlying health status, we calculated the Charlson Comorbidity Index (CCI) from ICD-10 codes recorded at hospital admission. The individual conditions comprising the CCI were also included as separate covariates in the original adjustment set. We also specifically flagged psychiatric diagnoses (depression, anxiety disorder, bipolar disorder) that could influence SSRI prescribing patterns [13]. During the index hospitalization, we identified claims-based diagnoses of acute organ dysfunction, including respiratory failure, renal failure, hepatic failure, shock, sepsis, coagulopathy/disseminated intravascular coagulation (DIC), and neurologic coma, using ICD-10 definitions [14]. These variables were used as claims-based indicators of acute illness severity. Recognizing that preexisting disability may affect both SSRI use and survival, we adjusted for disability status, which was obtained from the Korean National Disability Registration System. In South Korea, disability registration requires formal assessment by qualified specialists in the relevant clinical field after at least six months of persistent impairment, and disability severity is officially graded within this national framework [12]. Disability severity was categorized as severe for grades 1–3 and mild-to-moderate for grades 4–6 (Table S2). Lastly, to account for hospital-level and temporal factors, models included hospital classification (levels A, B, or C, reflecting resource availability), a binary indicator for surgery-associated admissions, and year of ICU admission (2020, 2021, 2022, or 2023) to control for evolving clinical practices and case‐mix over the study period.

### Statistical analyses

We summarized continuous variables as means with standard deviations and categorical variables as counts with percentages to describe the clinical and demographic characteristics of our cohort. Hospital classification was derived via hierarchical cluster analysis: we applied agglomerative clustering to facility-level metrics—including numbers of emergency room beds, operating room beds, ICU beds, total physicians, specialist physicians, nurses, and pharmacists—and identified three distinct groups (levels A, B, and C), the features of which are detailed in Table S3.

PSs were estimated using logistic regression. To mitigate confounding, we conducted 1:1 PS matching without replacement using the nearest-neighbor method and a caliper width of 0.10 × the standard deviation of the logit of the PS [15]. We assessed balance between SSRI and no SSRI prescription groups before and after matching using the absolute standardized mean difference (ASD), with an ASD < 0.10 indicating adequate balance. To further evaluate covariate balance, we also examined the distributional overlap of the estimated PSs before and after matching.

The primary analyses were performed using Cox proportional hazards models to estimate hazard ratios (HRs) and 95% confidence intervals (CIs) for 30-day and 90-day mortality. In the PS-matched cohort, Cox proportional hazards models were fitted with SSRI prescription as the primary independent variable. Kaplan–Meier survival curves with 95% CIs were generated for the matched cohort and displayed through 360 days after ICU admission to provide a longer-term visualization of survival patterns, with 90-day intervals shown on the time axis. Survival curves were compared using the log-rank test.

In the full, unmatched cohort, we fitted multivariable Cox proportional hazards models adjusting for covariates included in the PS model. For the additional post-hoc analysis, we identified patients whose recorded SSRI prescription supply period included the day immediately preceding ICU admission. Specifically, prescription coverage was considered present when the prescription start date was on or before index date −1 and the calculated prescription end date was on or after index date −1. The prescription end date was calculated as the prescription start date plus the number of days supplied minus one day. We then evaluated the association between SSRI prescription coverage through the day before ICU admission and 30-day and 90-day mortality using multivariable Cox proportional hazards models in the full cohort.

Subgroup analyses were performed according to the presence or absence of underlying psychiatric disorders. For the subgroup analysis, documented psychiatric disorders were operationally defined as recorded diagnoses of depression, anxiety disorder, or bipolar disorder. This definition was not intended to capture all psychiatric or nonpsychiatric indications for SSRI prescribing. To address potential site-level variation across heterogeneous ICUs, we performed stratified analyses according to hospital classification. The proportional hazards assumption was assessed using Schoenfeld residuals. All statistical tests were two-sided, conducted in R version 4.3.1, and used a significance threshold of P < 0.05.

## Results

### Study population

Between January 1, 2020, and December 31, 2023, we identified 1,690,346 ICU admission records in the NHIS database. After applying the eligibility criteria, including exclusion of pediatric admissions, repeat ICU admissions, and records with missing age or sex, the final analytic cohort comprised 1,189,042 adult patients with a first ICU admission during the study period (Fig. 1). Of these, 128,077 patients (10.8%) had an SSRI prescription supply overlapping the 30 days before ICU admission, whereas 1,060,965 patients (89.2%) had no SSRI prescription supply during this period. PS matching yielded 128,075 matched pairs for the primary outcome analyses. Table 1 presents the characteristics before and after PS matching. After matching, all absolute standardized differences were <0.10, indicating adequate covariate balance between the two groups. Notably, year of ICU admission was closely balanced after matching, with ASDs of 0.002, 0.003, 0.006, and 0.007 for 2020, 2021, 2022, and 2023, respectively. Figure S1 shows the distributions of PSs before and after matching. After matching, the PS distributions were more closely aligned between the SSRI and no SSRI prescription groups, supporting improved covariate overlap.

**Fig. 1:**
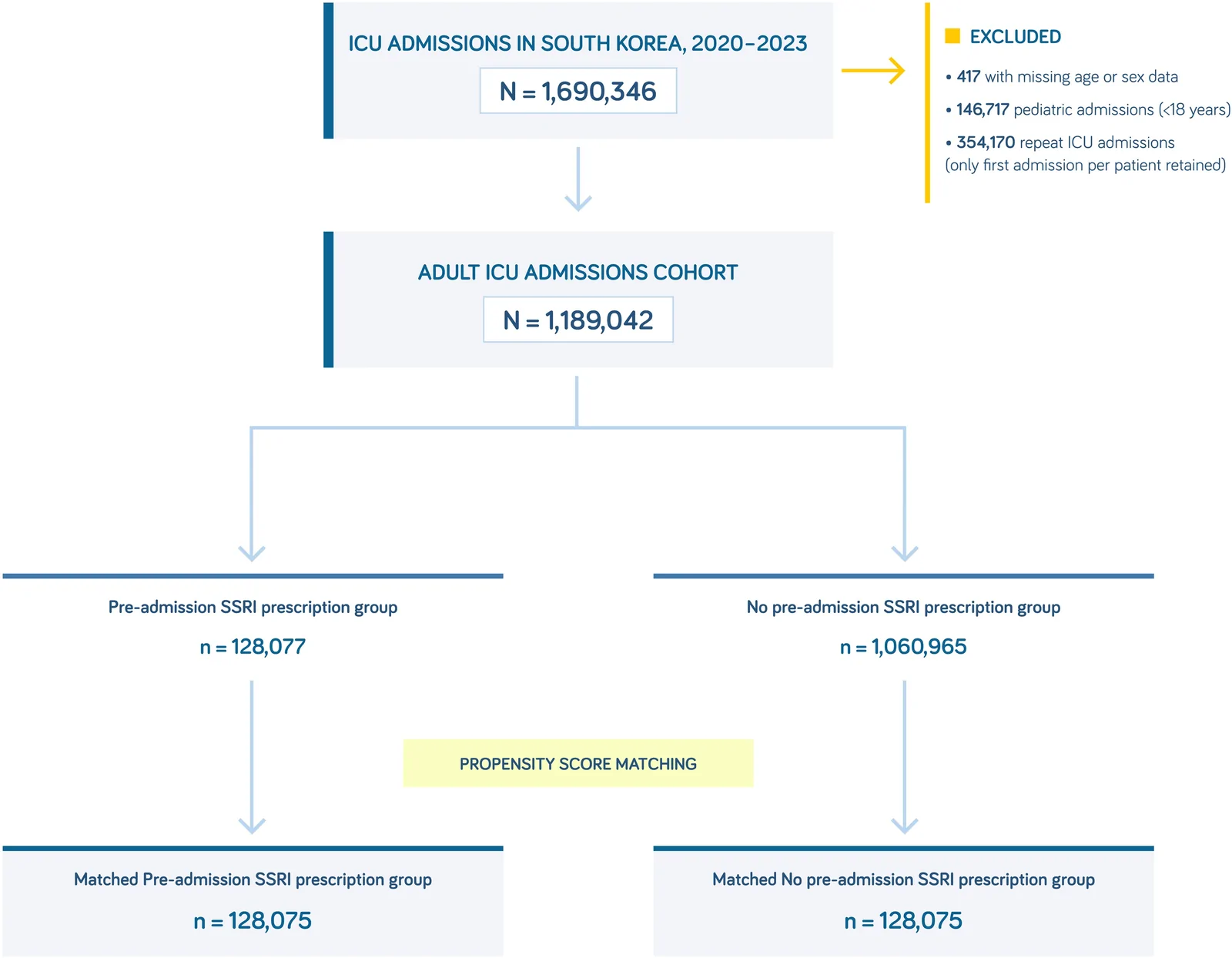
Flowchart depicting patient selection process. PS, propensity score; SSRI, Selective serotonin reuptake inhibitors; ICU, intensive care unit.

**Table 1:** Patient and admission characteristics before and after propensity score matching.

	Before PS matching (n = 1,189,042)		ASD	After PS matching (n = 256,150)		ASD
Variable	SSRI prescription group n = 128,077	No SSRI prescription group n = 1,060,965		SSRI prescription group n = 128,075	No SSRI prescription group n = 128,075	
Age, year	74.0 (14.3)	69.0 (15.5)	0.319	74.0 (14.3)	74.5 (14.2)	0.029
Female sex	71,230 (55.6)	437,614 (41.2)	0.260	71,228 (55.6)	72,747 (56.8)	0.024
Household income level						
Medical aid program group	19,417 (15.2)	109,078 (10.3)	0.032	19,415 (15.2)	19,869 (15.5)	0.008
Q1	24,317 (19.0)	214,659 (20.2)	0.062	24,317 (19.0)	23,933 (18.7)	0.008
Q2	17,241 (13.5)	169,080 (15.9)	0.052	17,241 (13.5)	16,910 (13.2)	0.002
Q3	23,052 (18.0)	215,645 (20.3)	0.020	23,052 (18.0)	22,966 (17.9)	0.008
Q4	42,384 (33.1)	336,702 (31.7)	0.125	42,384 (33.1)	42,834 (33.4)	0.010
Unknown	1,666 (1.3)	15,801 (1.5)	0.015	1,666 (1.3)	1,563 (1.2)	0.007
Residence						
Urban area	47,305 (36.9)	425,929 (40.1)		47,305 (36.9)	46,997 (36.7)	
Rural area	80,772 (63.1)	635,036 (59.9)	0.071	80,770 (63.1)	81,078 (63.3)	0.005
Underlying disability						
Mild to moderate	23,064 (18.0)	134,497 (12.7)	0.123	23,062 (18.0)	22,959 (17.9)	0.002
Severe	12,331 (9.6)	108,224 (10.2)	0.056	12,331 (9.6)	12,007 (9.4)	0.009
CCI, point	2.7 (2.3)	2.7 (2.4)	0.066	2.7 (2.3)	2.6 (2.3)	0.052
Myocardial infarction	8,829 (6.9)	106,464 (10.0)	0.097	8,829 (6.9)	8,130 (6.3)	0.022
Congestive heart failure	38,468 (30.0)	294,681 (27.8)	0.024	38,467 (30.0)	36,833 (28.8)	0.028
Peripheral vascular disease	8,175 (6.4)	66,154 (6.2)	0.011	8,174 (6.4)	7,833 (6.1)	0.011
Cerebrovascular disease	35,124 (27.4)	279,556 (26.3)	0.011	35,124 (27.4)	34,896 (27.2)	0.004
Dementia	17,731 (13.8)	64,917 (6.1)	0.228	17,729 (13.8)	16,485 (12.9)	0.029
Chronic pulmonary disease	32,600 (25.5)	244,430 (23.0)	0.031	32,599 (25.5)	31,226 (24.4)	0.025
Rheumatic disease	3,466 (2.7)	22,706 (2.1)	0.031	3,466 (2.7)	3,289 (2.6)	0.009
Peptic ulcer disease	13,654 (10.7)	119,436 (11.3)	0.011	13,654 (10.7)	12,887 (10.1)	0.020
Mild liver disease	23,896 (18.7)	204,922 (19.3)	0.043	23,896 (18.7)	22,489 (17.6)	0.029
DM without chronic complication	53,537 (41.8)	432,513 (40.8)	0.001	53,536 (41.8)	51,680 (40.4)	0.029
DM with chronic complication	6,415 (5.0)	45,891 (4.3)	0.016	6,415 (5.0)	6,072 (4.7)	0.012
Hemiplegia or paraplegia	5,179 (4.0)	49,044 (4.6)	0.035	5,179 (4.0)	5,080 (4.0)	0.004
Renal disease	9,756 (7.6)	91,895 (8.7)	0.072	9,756 (7.6)	9,134 (7.1)	0.019
Any malignancy	19,691 (15.4)	196,124 (18.5)	0.101	19,691 (15.4)	18,758 (14.6)	0.020
Moderate or severe liver disease	2,398 (1.9)	28,725 (2.7)	0.095	2,398 (1.9)	2,189 (1.7)	0.012
Metastatic solid tumour	3,864 (3.0)	38,931 (3.7)	0.055	3,864 (3.0)	3,691 (2.9)	0.008
AIDS/HIV	118 (0.1)	1,145 (0.1)	0.003	118 (0.1)	104 (0.1)	0.004
Documented psychiatric disorder						
Depression	21,659 (16.9)	51,151 (4.8)	0.319	21,657 (16.9)	20,051 (15.7)	0.034
Anxiety disorder	14,742 (11.5)	70,077 (6.6)	0.145	14,740 (11.5)	14,073 (11.0)	0.016
Bipolar disorder	12,582 (9.8)	62,194 (5.9)	0.125	12,582 (9.8)	11,909 (9.3)	0.018
Acute diagnoses recorded during the index hospitalization						
Respiratory failure	4,801 (3.7)	36,091 (3.4)	0.025	4,800 (3.7)	4,816 (3.8)	0.001
Renal failure	20,702 (16.2)	169,617 (16.0)	0.034	20,702 (16.2)	19,478 (15.2)	0.026
Hepatic failure	1,833 (1.4)	19,120 (1.8)	0.061	1,833 (1.4)	1,714 (1.3)	0.008
Shock	11,836 (9.2)	89,702 (8.5)	0.007	11,836 (9.2)	11,516 (9.0)	0.009
Sepsis	20,441 (16.0)	141,256 (13.3)	0.051	20,441 (16.0)	19,664 (15.4)	0.017
Coagulopathy / DIC	11,603 (9.1)	98,936 (9.3)	0.024	11,602 (9.1)	10,871 (8.5)	0.020
Neurologic Coma	626 (0.5)	3,812 (0.4)	0.005	626 (0.5)	610 (0.5)	0.002
Hospital level						
A	65,118 (50.8)	594,754 (56.4)	0.104	65,117 (50.8)	64,494 (50.4)	0.010
B	55,959 (43.7)	378,238 (35.7)	0.171	55,958 (43.7)	56,826 (44.4)	0.014
C	7,000 (5.5)	87,973 (8.3)	0.111	7,000 (5.5)	6,755 (5.3)	0.008
Surgery associated ICU admission	86,096 (67.2)	775,166 (73.1)	0.118	86,096 (67.2)	85,601 (66.8)	0.008
Year of ICU admission						
2020	27,036 (21.1)	284,575 (26.8)	0.140	27,036 (21.1)	26,922 (21.0)	0.002
2021	32,924 (25.7)	257,672 (24.3)	0.057	32,923 (25.7)	33,093 (25.8)	0.003
2022	34,636 (27.0)	256,197 (24.1)	0.066	34,636 (27.0)	34,981 (27.3)	0.006
2023	33,481 (26.1)	262,521 (24.7)	0.027	33,480 (26.1)	33,079 (25.8)	0.007

PS, propensity score; SSRI, Selective serotonin reuptake inhibitors; ASD, absolute standardized mean difference; CCI, Charlson comorbidity index; DM, diabetes mellitus; AIDS, Acquired immune deficiency syndrome; HIV, Human Immunodeficiency Virus; ICU, intensive care unit; DIC, Disseminated intravascular coagulation.

### Analyses in the PS-matched cohort

Table 2 summarizes the mortality outcomes before and after PS matching. In the matched cohort, 30-day mortality was 15.6% (19,957/128,075) in the SSRI prescription group and 16.2% (20,719/128,075) in the no SSRI prescription group. In Cox proportional hazards analysis, pre-ICU SSRI prescription was associated with a lower hazard of 30-day mortality (HR, 0.96; 95% CI, 0.94–0.98; *P* < 0.001). Ninety-day mortality was 25.3% (32,432/128,075) in the SSRI prescription group and 27.2% (34,796/128,075) in the no SSRI prescription group, corresponding to a lower hazard of 90-day mortality among patients with an SSRI prescription (HR, 0.93; 95% CI, 0.91–0.94; *P* < 0.001). Kaplan–Meier survival curves with 95% CIs are shown in Fig. 2. Survival differed significantly between the SSRI and no SSRI prescription groups by the log-rank test.

**Table 2:** Analyses before and after PS matching.

Outcome	Events, n/N (%)	HR (95% CI)	*P*-value
Before PS matching			
30-day mortality			
No SSRI prescription group	164,719/1,060,965 (15.5)	1	
SSRI prescription group	19,958/128,077 (15.6)	1.01 (0.99, 1.02)	0.461
90-day mortality			
No SSRI prescription group	267,702/1,060,965 (25.2)	1	
SSRI prescription group	32,433/128,077 (25.3)	1.00 (0.99, 1.02)	0.406
After PS matching			
30-day mortality			
No SSRI prescription group	20,719/128,075 (16.2)	1	
SSRI prescription group	19,957/128,075 (15.6)	0.96 (0.94, 0.98)	<0.001
90-day mortality			
No SSRI prescription group	34,796/128,075 (27.2)	1	
SSRI prescription group	32,432/128,075 (25.3)	0.93 (0.91, 0.94)	<0.001

HR, hazard ratio; CI, confidence interval; PS, propensity score; SSRI, Selective serotonin reuptake inhibitors.

**Fig. 2:**
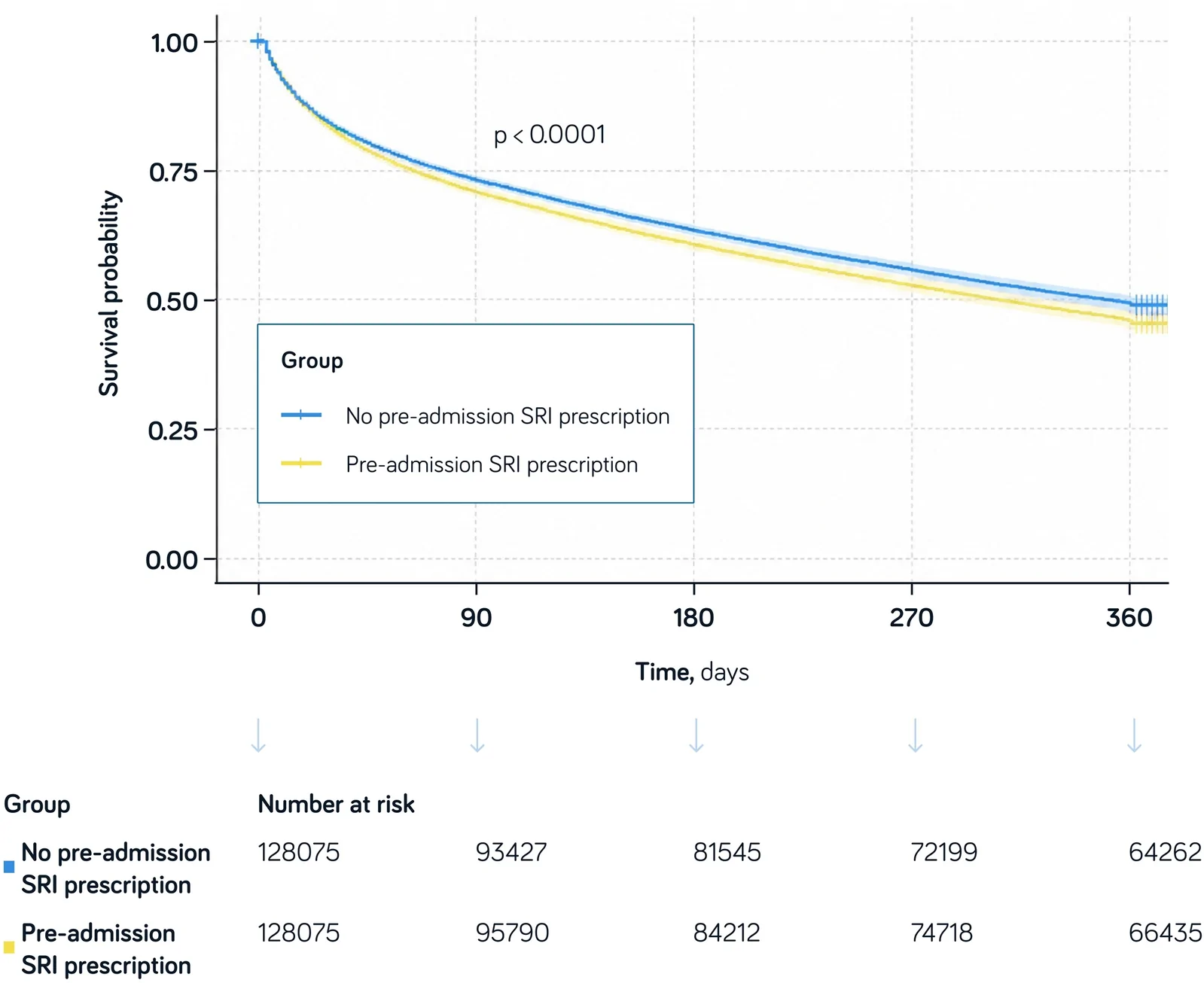
Kaplan–Meier survival curves for mortality after ICU admission in the propensity score-matched cohort. Curves are displayed through 360 days after ICU admission. ICU, intensive care unit; PS, propensity score; SSRI, selective serotonin reuptake inhibitor.

### Analyses in the entire cohort

Table 3 shows the results of multivariable Cox proportional hazards models in the full, unmatched cohort. After adjustment for covariates, pre-ICU SSRI prescription was associated with lower hazards of both 30-day mortality (HR, 0.96; 95% CI, 0.95–0.98; *P* < 0.001; model 1) and 90-day mortality (HR, 0.92; 95% CI, 0.91–0.93; *P* < 0.001; model 3). In the additional post-hoc analysis of pre-ICU SSRI prescription, 78,250 patients had SSRI prescription supply overlapping the day immediately before ICU admission. This more restrictive prescription definition was also associated with lower hazards of 30-day mortality (HR, 0.93; 95% CI, 0.92–0.94; *P* < 0.001; model 2) and 90-day mortality (HR, 0.90; 95% CI, 0.89–0.91; *P* < 0.001; model 4). The complete covariate estimates for the primary 30-day and 90-day models (models 1 and 3) are provided in Tables S4 and S5, respectively.

**Table 3:** Multivariable Cox regression in the full, unmatched cohort.

Outcome	HR (95% CI)	*P*-value
30-day mortality, model 1		
No SSRI prescription group	1	
SSRI prescription group	0.96 (0.95, 0.98)	<0.001
30-day mortality, model 2		
No SSRI prescription group	1	
SSRI prescription coverage through the day before ICU admission (n = 78,250)	0.93 (0.92, 0.94)	<0.001
90-day mortality, model 3		
No SSRI prescription group	1	
SSRI prescription group	0.92 (0.91, 0.93)	<0.001
90-day mortality, model 4		
No SSRI prescription group	1	
SSRI prescription coverage through the day before ICU admission (n = 78,250)	0.90 (0.89, 0.91)	<0.001

HR, hazard ratio; CI, confidence interval; SSRI, Selective serotonin reuptake inhibitors.

### Subgroup analyses

Subgroup analyses stratified by the presence of underlying psychiatric disorders are presented in Table 4. Among patients with documented psychiatric disorders (n = 190,369), pre-ICU SSRI prescription was associated with lower hazards of 30-day mortality (HR, 0.84; 95% CI, 0.81–0.87; *P* < 0.001) and 90-day mortality (HR, 0.79; 95% CI, 0.76–0.81; *P* < 0.001). Among patients without documented psychiatric disorders (n = 998,673), the corresponding associations were more modest but remained statistically significant for both 30-day mortality (HR, 0.97; 95% CI, 0.96–0.98; *P* = 0.001) and 90-day mortality (HR, 0.91; 95% CI, 0.90–0.93; *P* < 0.001).

**Table 4:** Subgroup analyses.

Outcome	HR (95% CI)	*P-*value
Documented psychiatric disorder group (n = 190,369)		
30-day mortality		
No SSRI prescription group	1	
SSRI prescription group	0.84 (0.81, 0.87)	<0.001
90-day mortality		
No SSRI prescription group	1	
SSRI prescription group	0.79 (0.76, 0.81)	<0.001
No documented psychiatric disorder group (n = 998,673)		
30-day mortality		
No SSRI prescription group	1	
SSRI prescription group	0.97 (0.96, 0.98)	0.001
90-day mortality		
No SSRI prescription group	1	
SSRI prescription group	0.91 (0.90, 0.93)	<0.001
Hospital level A group (n = 659,872)		
30-day mortality		
No SSRI prescription group	1	
SSRI prescription group	0.90 (0.88, 0.92)	<0.001
90-day mortality		
No SSRI prescription group	1	
SSRI prescription group	0.89 (0.87, 0.90)	<0.001
Hospital level B group (n = 434,197)		
30-day mortality		
No SSRI prescription group	1	
SSRI prescription group	0.86 (0.84, 0.88)	<0.001
90-day mortality		
No SSRI prescription group	1	
SSRI prescription group	0.86 (0.84, 0.87)	<0.001
Hospital level C group (n = 94,973)		
30-day mortality		
No SSRI prescription group	1	
SSRI prescription group	0.85 (0.80, 0.91)	<0.001
90-day mortality		
No SSRI prescription group	1	
SSRI prescription group	0.85 (0.80, 0.91)	<0.001

Documented psychiatric disorders were defined as depression, anxiety disorder, or bipolar disorder.

HR, hazard ratio; CI, confidence interval; SSRI, Selective serotonin reuptake inhibitors.

In analyses stratified by hospital classification, pre-ICU SSRI prescription was consistently associated with lower hazards of mortality across hospital levels A, B, and C. For 30-day mortality, the HRs were 0.90 (95% CI, 0.88–0.92) in level A hospitals, 0.86 (95% CI, 0.84–0.88) in level B hospitals, and 0.85 (95% CI, 0.80–0.91) in level C hospitals. For 90-day mortality, the corresponding HRs were 0.89 (95% CI, 0.87–0.90), 0.86 (95% CI, 0.84–0.87), and 0.85 (95% CI, 0.80–0.91), respectively.

## Discussion

In this large, nationwide cohort of critically ill adults, pre-ICU SSRI prescription was associated with lower short- and intermediate-term mortality after PS matching and multivariable adjustment. This association was also observed in the additional post-hoc analysis restricted to patients with SSRI prescription supply up to the day immediately before ICU admission. The estimated association was numerically stronger among patients with documented psychiatric disorders, although these subgroup findings should be interpreted cautiously because psychiatric diagnoses were identified using administrative claims codes. Overall, the observed association was consistent across the available primary and additional analyses, but the observational design and limitations of claims-based covariate adjustment preclude causal interpretation.

Several biological mechanisms may plausibly link SSRI prescription to outcomes in severe illness, although these mechanisms remain speculative in the context of our observational study. One plausible mechanism by which SSRIs may improve survival in critical illness is through an interleukin-10 (IL‑10)–dependent immunometabolic pathway originally described for fluoxetine in experimental sepsis [3]. In murine models, fluoxetine pretreatment increased circulating IL-10 levels independent of peripheral serotonin signaling, and IL-10 was associated with attenuation of sepsis-induced metabolic disturbances and organ dysfunction [3]. These preclinical findings provide biological plausibility for an association between SSRI prescription and survival in critical illness, but whether similar mechanisms operate in humans with heterogeneous ICU diagnoses remains uncertain.

SSRIs may also influence inflammatory, endothelial, platelet, and cellular stress-response pathways. Some SSRIs, including fluvoxamine, sertraline, and fluoxetine, have activity at the endoplasmic-reticulum σ1 receptor, which may modulate cellular stress responses and inflammatory signaling [16]. In addition, several SSRIs have functional acid sphingomyelinase inhibitory activity, a mechanism that has been implicated in ceramide-mediated endothelial inflammation and host responses during severe infection [17]. SSRIs have also been reported to affect proinflammatory signaling pathways, including NF-κB activation and NLRP3 inflammasome activity, and to inhibit platelet serotonin uptake and aggregation [18,19]. These mechanisms could theoretically influence outcomes in critical illness, but our claims-based study was not designed to directly evaluate biological pathways, drug-specific pharmacodynamics, in-hospital continuation, or dose-response relationships.

The stronger association observed among patients with underlying psychiatric disorders requires careful interpretation. One possible explanation is that patients with psychiatric disorders may be more likely to have sustained SSRI prescription before ICU admission or may experience adverse effects from abrupt SSRI discontinuation during critical illness. SSRI discontinuation has been discussed in relation to delirium and neuropsychiatric complications in critically ill patients, and delirium itself is an important prognostic factor in the ICU [20]. In addition, antidepressant treatment may be related to patient resilience, motivation, and participation in rehabilitation during the early post-ICU period. These explanations are not mutually exclusive and may be particularly relevant in patients with preexisting psychiatric disorders. However, because psychiatric comorbidity was defined using ICD-10 codes and because information on in-ICU medication continuation, delirium, rehabilitation intensity, and patient motivation was unavailable, these interpretations should be considered hypothesis-generating. Moreover, the subgroup without these documented psychiatric diagnoses should not be interpreted as representing patients with no psychiatric condition or no clinical indication for SSRI treatment. This group may have included patients with unrecorded or incompletely coded psychiatric disorders, other psychiatric indications not included in our operational definition, or nonpsychiatric indications for SSRI therapy.

Our findings should also be interpreted in the context of prior literature, which remains mixed. Earlier ICU-based observational studies reported higher mortality among pre-ICU SSRI users, whereas studies in COVID-19 populations suggested potential benefits of selected SSRIs such as fluvoxamine or fluoxetine in specific clinical settings [[4], [5], [6], [7], [8], [9]]. Differences in study populations, timing of prescription, specific SSRI agents, indications for treatment, outcome definitions, and adjustment for confounding may partly explain these discrepancies. By using a nationwide ICU cohort, PS matching with a narrower caliper, Cox proportional hazards models, and an additional analysis based on prescription coverage through the day before ICU admission, our study adds population-level evidence on the association between pre-ICU SSRI prescription and mortality. Nevertheless, the observational design precludes causal inference, and the results should not be interpreted as evidence that initiating or continuing SSRIs improves ICU outcomes.

This study has several important limitations. First, its observational design cannot establish causality, and residual confounding may persist despite PS matching, multivariable adjustment, and additional diagnostic checks. In particular, physiologic severity scores such as Acute Physiology and Chronic Health Evaluation II, Sequential Organ Failure Assessment, or Simplified Acute Physiology Score were not available in the NHIS claims database. We therefore adjusted for claims-based indicators of acute organ dysfunction and institutional factors, but these variables may not fully capture illness severity. Second, SSRI prescription was inferred from prescription records and may not perfectly reflect actual medication adherence or in‑hospital continuation; information on dose, formulation, and in‑ICU administration was unavailable. Third, psychiatric comorbidity was defined using diagnostic codes, which may undercapture subclinical, undiagnosed, or incompletely coded conditions and may therefore affect the subgroup analyses. Confounding by indication is also an important consideration because depression, anxiety disorder, and bipolar disorder may influence both SSRI prescribing and clinical outcomes. Claims data do not capture psychiatric disease severity, treatment response, duration of illness, or the specific indication for SSRI prescription. Consequently, adjustment for diagnostic categories alone may not fully account for indication-related confounding and, depending on the underlying causal structure, could also introduce bias. The stronger association observed among patients with documented psychiatric disorders should therefore be interpreted cautiously. Fourth, the CCI summary score and its constituent comorbidities were simultaneously included in the original covariate adjustment set. Although the individual comorbidity coefficients were not outcomes of interest and were not interpreted as independent prognostic effects, this parameterization may complicate interpretation of the individual covariate estimates. Alternative model specifications using either the summary CCI score or its individual components were not evaluated. In addition, the CCI was derived from diagnoses recorded at hospital admission rather than from an exclusively pre-index lookback window, which may have limited distinction between preexisting comorbidity and conditions coded during the index hospitalization. Fifth, acute organ dysfunction variables were identified during the index hospitalization rather than exclusively before ICU admission. Accordingly, some of these variables may have occurred after the pre-admission prescription assessment and may lie on or near the pathway between pre-admission factors and mortality. Adjustment for these variables could therefore introduce overadjustment or conditioning bias, and the corresponding multivariable estimates should be interpreted with this limitation in mind. Sixth, the Cox models in the PS-matched cohort did not explicitly account for within-pair dependence using matched-pair stratification or a cluster-robust variance estimator. Consequently, the precision of the reported confidence intervals may be affected. Seventh, we did not separately identify COVID-19-related ICU admissions or pandemic waves; therefore, residual confounding related to changes in pandemic burden and ICU case mix during 2020–2023 cannot be excluded. Eighth, the calendar-year coefficients in the fully adjusted models were unexpectedly large, particularly for 2023, and should not be interpreted as estimates of secular changes in ICU mortality. Because only the first ICU admission during 2020–2023 was retained, eligibility in later years became progressively conditional on having had no prior ICU admission during the study period, potentially altering case mix across calendar years. This design feature may have contributed to the large calendar-year coefficients, although its effect could not be quantified. Importantly, year of ICU admission was closely balanced after PS matching, and the association between SSRI prescription and mortality remained present in the matched cohort. Finally, although the cohort reflects nationwide practice in South Korea, the findings may not be generalizable to healthcare systems with different ICU admission practices, prescribing patterns, or medication continuation protocols.

## Conclusions

In this nationwide cohort of critically ill adults, pre-admission SSRI prescription was associated with lower 30-day and 90-day mortality. Similar findings were observed among patients whose prescription supply covered the day immediately before ICU admission. However, the observational design, potential residual and indication-related confounding, and limitations of claims-based covariate adjustment preclude causal inference. These findings should therefore be considered hypothesis-generating.

## Authors' contributions

T.K.O. and I.-A.S. conceived and designed the study. T.K.O. performed data extraction, statistical analyses, and drafted the manuscript. I.-A.S. supervised the analyses, interpreted results, and critically revised the manuscript. All authors read and approved the final manuscript.

## Consent for publication

Not applicable.

## Ethics approval and consent to participate

This study was approved by the Institutional Review Board of Seoul National University Bundang Hospital (X‑2410‑930‑905) and the National Health Insurance Service (NHIS‑2025‑04‑1‑108). As all data were fully anonymized and originally collected for routine administrative purposes, the requirement for individual informed consent was waived in accordance with Korean regulations and the Declaration of Helsinki.

## Funding

No external funding was received for this study.

## Availability of data and materials

The data used in this study are not publicly available. Access to individual-level NHIS data is granted only within an approved secure research environment for a predefined access period and requires separate authorization from the NHIS. Investigators are not permitted to retain individual-level data after expiration of the approved access period.

## Declaration of competing interest

The authors declare that they have no competing interests.

## Figures and Tables

**Figure:**