# Supplementary material for: Pre-admission prescription of selective serotonin reuptake inhibitors and mortality in critical illness: a national cohort study from 2020 to 2023
Source: Ann Intensive Care. 2026 Sep 17;16:100161. doi: 10.1016/j.aicoj.2026.100161 (PMC13634290; doi:10.1016/j.aicoj.2026.100161)
Supplement: Supplementary file 3 [file mmc3.docx]

Table S3. Hospital features

| Variable | | Type A  n=81 | Type B  n=332 | Type C  n=4 |
| --- | --- | --- | --- | --- |
| Type of hospital | |  |  |  |
|  | Tertiary general hospital | 40 (49.4) | 0 (0.0) | 4 (100.0) |
|  | General hospital | 39 (48.1) | 253 (76.2) | 0 (0.0) |
|  | Other hospital | 2 (2.5) | 79 (23.8) | 0 (0.0) |
| Total number of hospital bed, n (SD) | | 710.1 (170.8) | 239.3 (125.3) | 1,945.5 (413.1) |
| Total number of adult ICU bed, n (SD) | | 46.4 (17.4) | 12.3 (10.5) | 75.8 (6.7) |
| Total number of OR bed, n (SD) | | 17.2 (4.8) | 3.9 (2.5) | 61.0 (13.8) |
| Total number of ER bed, n (SD) | | 29.6 (7.9) | 11.3 (7.5) | 63.0 (30.1) |
| Total number of doctor, n (SD) | | 317.7 (140.6) | 37.9 (36.9) | 1,413.0 (194.5) |
| Total number of specialist doctors, n (SD) | | 195.8 (78.0) | 33.1 (26.6) | 864.8 (92.8) |
| Total number of nurse, n (SD) | | 1,005.7 (377.7) | 190.4 (140.0) | 3,582.5 (539.1) |
| Total number of pharmacist | | 27.4 (15.4) | 2.8 (3.4) | 138.3 (23.6) |

SD, standard deviation; ICU, intensive care unit; OR, operating room; ER, emergency room.
