# Supplementary material for: Pre-admission prescription of selective serotonin reuptake inhibitors and mortality in critical illness: a national cohort study from 2020 to 2023
Source: Ann Intensive Care. 2026 Sep 17;16:100161. doi: 10.1016/j.aicoj.2026.100161 (PMC13634290; doi:10.1016/j.aicoj.2026.100161)
Supplement: Supplementary file 4 [file mmc4.docx]

Table S4. The complete set of adjusted HRs and 95% CIs for all covariates in model 1

| Variable | | HR (95% CI) | *P*-value |
| --- | --- | --- | --- |
| Age, year | | 1.03 (1.03, 1.03) | <0.001 |
| Female sex | | 0.87 (0.86, 0.88) | <0.001 |
| Household income level | |  |  |
|  | Medical aid program group | 1.04 (1.03, 1.06) | <0.001 |
|  | Q1 | 1 |  |
|  | Q2 | 0.99 (0.98, 1.01) | 0.265 |
|  | Q3 | 0.96 (0.95, 0.98) | <0.001 |
|  | Q4 | 0.95 (0.94, 0.96) | <0.001 |
|  | Unknown | 1.01 (0.96, 1.05) | 0.824 |
| Residence | |  |  |
|  | Urban area | 1 |  |
|  | Rural area | 0.97 (0.96, 0.98) | <0.001 |
| Underlying disability | |  |  |
|  | Mild to moderate | 0.95 (0.94, 0.97) | <0.001 |
|  | Severe | 1.10 (1.09, 1.12) | <0.001 |
| CCI, point | | 1.06 (1.06, 1.06) | <0.001 |
|  | Myocardial infarction | 0.97 (0.96, 0.99) | 0.001 |
|  | Congestive heart failure | 1.09 (1.08, 1.10) | <0.001 |
|  | Peripheral vascular disease | 0.76 (0.74, 0.77) | <0.001 |
|  | Cerebrovascular disease | 1.12 (1.11, 1.13) | <0.001 |
|  | Dementia | 0.70 (0.69, 0.71) | <0.001 |
|  | Chronic pulmonary disease | 1.09 (1.08, 1.10) | <0.001 |
|  | Rheumatic disease | 0.92 (0.89, 0.94) | <0.001 |
|  | Peptic ulcer disease | 0.85 (0.84, 0.86) | <0.001 |
|  | Mild liver disease | 1.07 (1.05, 1.08) | <0.001 |
|  | Diabetes without chronic complication | 0.95 (0.94, 0.96) | <0.001 |
|  | Diabetes with chronic complication | 0.75 (0.74, 0.77) | <0.001 |
|  | Hemiplegia or paraplegia | 0.57 (0.55, 0.59) | <0.001 |
|  | Renal disease | 0.73 (0.72, 0.74) | <0.001 |
|  | Any malignancy | 1.19 (1.18, 1.20) | <0.001 |
|  | Moderate or severe liver disease | 2.08 (2.01, 2.15) | <0.001 |
|  | Metastatic solid tumour | 1.41 (1.38, 1.44) | <0.001 |
|  | AIDS/HIV | 0.99 (0.87, 1.13) | 0.867 |
| Documented psychiatric disorders | |  |  |
|  | Depression | 0.60 (0.59, 0.61) | <0.001 |
|  | Anxiety disorder | 0.81 (0.80, 0.83) | <0.001 |
|  | Bipolar disorder | 0.58 (0.57, 0.59) | <0.001 |
| Acute diagnoses recorded during the index hospitalization | |  |  |
|  | Respiratory failure | 2.54 (2.48, 2.59) | <0.001 |
|  | Renal failure | 2.12 (2.09, 2.14) | <0.001 |
|  | Hepatic failure | 1.09 (1.04, 1.15) | <0.001 |
|  | Shock | 1.93 (1.89, 1.96) | <0.001 |
|  | Sepsis | 1.65 (1.63, 1.68) | <0.001 |
|  | Coagulopathy / DIC | 1.51 (1.49, 1.54) | <0.001 |
|  | Neurologic Coma | 2.23 (2.13, 2.34) | <0.001 |
| Hospital level | |  |  |
|  | A | 1 |  |
|  | B | 1.12 (1.11, 1.14) | <0.001 |
|  | C | 0.60 (0.58, 0.61) | <0.001 |
| Surgery associated ICU admission | | 0.97 (0.95, 0.98) | <0.001 |
| Year of ICU admission | |  |  |
|  | 2020 | 1 |  |
|  | 2021 | 0.96 (0.94, 0.97) | <0.001 |
|  | 2022 | 1.09 (1.07, 1.10) | <0.001 |
|  | 2023 | 1.65 (1.63, 1.68) | <0.001 |

HR, hazard ratio; CI, confidence interval; CCI, Charlson comorbidity index; AIDS, Acquired immune deficiency syndrome; HIV, Human Immunodeficiency Virus; ICU, intensive care unit; DIC, Disseminated intravascular coagulation
