# Supplementary material for: Pre-admission prescription of selective serotonin reuptake inhibitors and mortality in critical illness: a national cohort study from 2020 to 2023
Source: Ann Intensive Care. 2026 Sep 17;16:100161. doi: 10.1016/j.aicoj.2026.100161 (PMC13634290; doi:10.1016/j.aicoj.2026.100161)
Supplement: Supplementary file 5 [file mmc5.docx]

Table S5. The complete set of adjusted HRs and 95% CIs for all covariates in model 3

| Variable | | HR (95% CI) | *P*-value |
| --- | --- | --- | --- |
| Age, year | | 1.03 (1.03, 1.03) | <0.001 |
| Female sex | | 0.83 (0.82, 0.84) | <0.001 |
| Household income level | |  |  |
|  | Medical aid program group | 1.09 (1.07, 1.11) | <0.001 |
|  | Q1 | 1 |  |
|  | Q2 | 0.99 (0.97, 1.01) | 0.177 |
|  | Q3 | 0.96 (0.95, 0.98) | <0.001 |
|  | Q4 | 0.94 (0.92, 0.95) | <0.001 |
|  | Unknown | 1.07 (1.03, 1.11) | 0.001 |
| Residence | |  |  |
|  | Urban area | 1 |  |
|  | Rural area | 0.98 (0.97, 0.99) | <0.001 |
| Underlying disability | |  |  |
|  | Mild to moderate | 0.94 (0.93, 0.96) | <0.001 |
|  | Severe | 1.07 (1.05, 1.08) | <0.001 |
| CCI, point | | 1.08 (1.08, 1.09) | <0.001 |
|  | Myocardial infarction | 0.89 (0.88, 0.91) | <0.001 |
|  | Congestive heart failure | 1.09 (1.08, 1.10) | <0.001 |
|  | Peripheral vascular disease | 0.81 (0.79, 0.82) | <0.001 |
|  | Cerebrovascular disease | 1.16 (1.15, 1.17) | <0.001 |
|  | Dementia | 0.89 (0.87, 0.91) | <0.001 |
|  | Chronic pulmonary disease | 1.19 (1.17, 1.20) | <0.001 |
|  | Rheumatic disease | 0.99 (0.96, 1.02) | 0.524 |
|  | Peptic ulcer disease | 0.88 (0.86, 0.89) | <0.001 |
|  | Mild liver disease | 1.07 (1.05, 1.08) | <0.001 |
|  | Diabetes without chronic complication | 0.94 (0.93, 0.95) | <0.001 |
|  | Diabetes with chronic complication | 0.80 (0.78, 0.82) | <0.001 |
|  | Hemiplegia or paraplegia | 0.68 (0.66, 0.69) | <0.001 |
|  | Renal disease | 0.75 (0.73, 0.76) | <0.001 |
|  | Any malignancy | 1.46 (1.44, 1.48) | <0.001 |
|  | Moderate or severe liver disease | 2.51 (2.41, 2.61) | <0.001 |
|  | Metastatic solid tumour | 1.93 (1.88, 1.97) | <0.001 |
|  | AIDS/HIV | 0.97 (0.85, 1.11) | 0.668 |
| Documented psychiatric disorders | |  |  |
|  | Depression | 0.75 (0.74, 0.77) | <0.001 |
|  | Anxiety disorder | 0.87 (0.86, 0.89) | <0.001 |
|  | Bipolar disorder | 0.77 (0.76, 0.79) | <0.001 |
| Acute diagnoses recorded during the index hospitalization | |  |  |
|  | Respiratory failure | 2.92 (2.86, 2.99) | <0.001 |
|  | Renal failure | 2.42 (2.38, 2.46) | <0.001 |
|  | Hepatic failure | 1.11 (1.06, 1.16) | <0.001 |
|  | Shock | 1.76 (1.73, 1.79) | <0.001 |
|  | Sepsis | 1.68 (1.66, 1.76) | <0.001 |
|  | Coagulopathy / DIC | 1.45 (1.43, 1.47) | <0.001 |
|  | Neurologic Coma | 2.50 (2.34, 2.67) | <0.001 |
| Hospital level | |  |  |
|  | A | 1 |  |
|  | B | 1.14 (1.13, 1.15) | <0.001 |
|  | C | 0.71 (0.70, 0.73) | <0.001 |
| Surgery associated ICU admission | | 1.01 (1.00, 1.02) | 0.015 |
| Year of ICU admission | |  |  |
|  | 2020 | 1 |  |
|  | 2021 | 0.97 (0.96, 0.98) | <0.001 |
|  | 2022 | 1.09 (1.08, 1.11) | <0.001 |
|  | 2023 | 2.92 (2.89, 2.96) | <0.001 |

HR, hazard ratio; CI, confidence interval; CCI, Charlson comorbidity index; AIDS, Acquired immune deficiency syndrome; HIV, Human Immunodeficiency Virus; ICU, intensive care unit; DIC, Disseminated intravascular coagulation
